# Metabolic engineering of vitamin D_3_ in *Solanaceae* plants

**DOI:** 10.1111/pbi.14459

**Published:** 2024-09-16

**Authors:** Marianna Boccia, Kerstin Ploβ, Maritta Kunert, Radhika Keshan, Mustafa Hatam, Veit Grabe, Sarah E. O'Connor, Prashant D. Sonawane

**Affiliations:** ^1^ Department of Natural Product Biosynthesis Max Planck Institute for Chemical Ecology Jena Germany; ^2^ Microscopy Imaging Service Max Planck Institute for Chemical Ecology Jena Germany

**Keywords:** 7‐dehydrocholesterol, vitamin D_3_, tomato, metabolic engineering, gene editing

Vitamin D is a lipid‐soluble sterol that plays an essential role in human health. Deficiency of this vitamin increases the risk of osteoporosis, hypertension, autoimmune diseases, infectious disease, diabetes and cancer. Vitamin D exists in two major forms: vitamin D_3_ (cholecalciferol), mainly found in animal food source, and vitamin D_2_ (ergocalciferol), typically present in sundried and ultraviolet‐B (UV‐B) exposed fungi and yeast (Jäpelt *et al*., 2013). Vitamin D_3_ is produced in human skin upon sunlight exposure, where pro‐vitamin D_3_ (7‐dehydrocholesterol; 7‐DHC) is converted to vitamin D_3_ by UV‐B light (290–315 nm). Unfortunately, vitamin D_3_ deficiency is common in both children and adults worldwide. Endogenous synthesis of vitamin D_3_ in human skin is inhibited by several factors such as melanin presence, sunlight intensity, pollution and geographic location. Therefore, dietary sources are essential for maintaining consistent vitamin D_3_ levels. Unfortunately, few dietary sources and supplements naturally contain vitamin D_3_ and most of these are animal‐based foods (e.g. meat and eggs), which raises concerns about vitamin D_3_ levels among those populations that consume low amounts of animal products (Black *et al*., 2017).

Plants harbour an enormous reservoir of diverse steroidal molecules and, in principle, could be a source of vitamin D_3_. However, although vitamin D_3_ has been identified in some plants and algae, the levels are much lower compared to animal‐based sources. The precursor of vitamin D_3_, 7‐DHC, is also the immediate precursor for cholesterol biosynthesis in plants (Figure 1a) (Sonawane *et al*., 2017). Since most plants produce cholesterol in very low amounts, 7‐DHC levels are low as well. Notably, *Solanaceae* family members (e.g. tomato and *Nicotiana benthamiana*) accumulate naturally high levels of cholesterol. In tomato and other *Solanum* food crops such as potato and eggplant, cholesterol serves as a starting precursor for biosynthesis of defensive steroidal glycoalkaloids (SGAs) (Sonawane *et al*., 2017). Using the recently elucidated cholesterol pathway in plants along with gene editing strategies, it is now possible to engineer high levels of 7‐DHC and therefore, vitamin D_3_ in plants. Here, we report metabolic engineering approaches to enhance vitamin D_3_ production in tomato (*Solanum lycopersicum*) and *N. benthamiana* plants.

**Figure 1:**
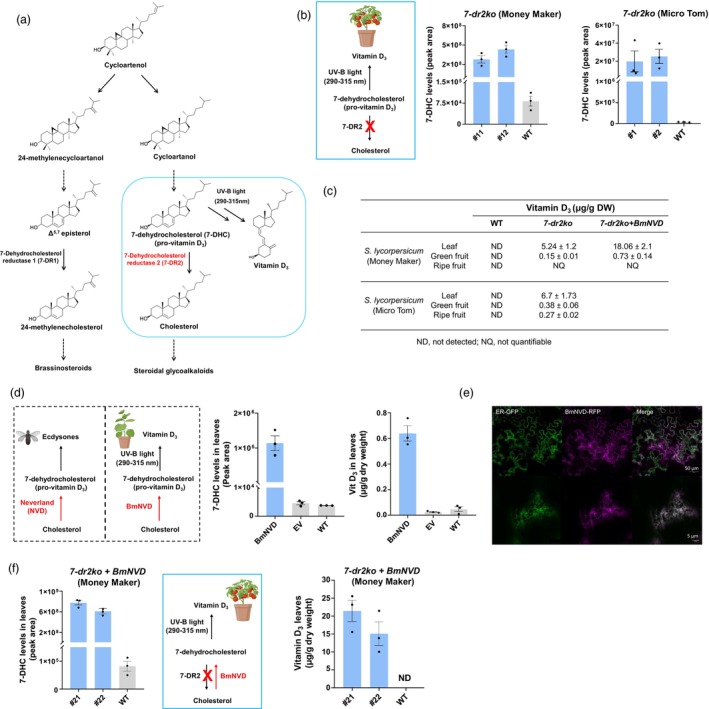
Overproduction of vitamin D_3_ in tomato and *N. benthamiana* plants using various genetic engineering strategies. (a) Cholesterol (right) and phytosterol (left) biosynthetic pathways in tomato starting from the cycloartenol precursor. The sterol 7‐DHC, a key intermediate in the cholesterol pathway, can be converted to vitamin D_3_ by exposure to UV‐B light. Solid and dashed arrows suggest single or multiple enzymatic steps in the pathway, respectively. (b) Levels of 7‐DHC in leaves of wild type (WT) and *7‐dr2ko S. lycopersicum cv*. Money Maker (left) and *cv*. Micro Tom (right) mutant lines as determined by GC–MS. The values indicate means of biological replicates ± standard error mean (*n* = 3 biological replicates obtained either from WT or independent mutant line). (c) Average yields of vitamin D_3_ produced in leaves, green fruit and ripe fruit of WT, *7‐dr2ko* and *BmNVD + 7‐dr2ko* genotypes upon exposure to UV‐B light. (d) Transient overexpression of *Bombyx mori Neverland* (*BmNVD*, codon optimized) in *Nicotiana benthamiana* leaves. Levels of 7‐DHC and vitamin D_3_ in *N. benthamiana* leaves that transiently express *BmNVD* compared to empty vector control and wild type. (e) Confocal microscopy images showing the sub‐cellular localization of BmNVD. Red fluorescent protein (RFP) was employed as a reporter and fused to the C‐terminus of BmNVD and co‐infiltrated along with an endoplasmic reticulum cellular compartment marker (AtUBI10:GFP‐HDEL), in 3‐week‐old *N. benthamiana* leaves. Each panel (upper and lower) contains images of RFP, GFP, and both channel merged. Scale bars: upper panels 50 μm, lower panels 5 μm. (f) Levels of 7‐DHC in and vitamin D_3_ in leaves of WT and *7‐dr2ko + BmNVD S. lycopersicum cv*. Money Maker lines as determined by GC–MS. The values indicate means of biological replicates ± standard error mean (*n* = 3). ND, not detected.

The sterol 7‐dehydrocholesterol (7‐DHC) is an intermediate in the cholesterol biosynthetic pathway and is converted to vitamin D_3_ upon sunlight exposure. Thus, overproduction of vitamin D_3_ in plants requires a significant accumulation of 7‐DHC. In *Solanaceae* plants, for example tomato, where high levels of cholesterol are produced, two related sterol‐Δ^7^ reductase enzymes (7‐dehydrocholesterol reductases; 7‐DR1 and 7‐DR2, 80% amino acid identity) were identified. Moreover, 7‐DR1/DWARF5 catalyses the reduction of pathway intermediates (e.g. Δ^5,7^‐episterol) in phytosterols biosynthesis, while 7‐DR2, which evolved from 7‐DR1 through duplication and divergence, produces cholesterol from 7‐DHC (Figure 1a) (Sonawane *et al*., 2017). Thus, the *7‐DR2* gene is an ideal target for altering cholesterol metabolism and 7‐DHC accumulation can be achieved by genome editing of *7‐DR2* in tomato. Recently, Li *et al*. (2022) reported the production of vitamin D_3_ in tomato (*S. lycopersicum cv*. Money Maker) by knocking out the *7‐dr2* gene (*7‐dr2ko*), achieving vitamin D_3_ yields of 200 μg/g dry weight (DW) in leaves, 0.3 μg/g DW in green fruit and 0.2 μg/g DW in red fruit. We also generated the loss‐of‐function *7‐dr2* mutants in two tomato varieties (*cv*. Money Maker and *cv*. Micro Tom) using CRISPR‐Cas9 genome editing (Figures S1a and S2a). Loss of function of *7‐dr2* had no visible effect on the growth, development and fruit yield of the mutant lines as compared to wild type (WT) plants (Figure S2b). Leaves and fruits from homozygous 7*‐dr2* mutant lines were analysed for 7‐DHC and cholesterol metabolites using gas chromatography–mass spectrometry (GC–MS) (Figure 1B; Figures S1b,c,e,f and S2c,d). In both cultivars, the *7‐dr2* mutant leaves and green fruits showed accumulation of 7‐DHC, the expected product. Treatment of these tissues with UV‐B light for 30 min with an intensity of 22 W/m^2^ resulted in vitamin D_3_ yields in Money Maker of 5.24 ± 1.2 μg/g of DW in leaves and 0.15 ± 0.01 μg/g in green fruit and no quantifiable levels in ripe fruit (Figure 1c; Figure S1d,g). In the previous study, Li *et al*. reported higher levels of vitamin D_3_ in Money Maker mutant plant tissues, particularly in the leaves, despite targeting the same gene knockout. We hypothesize that this discrepancy could be attributable to differences in the UV‐B treatment, which was performed for 1 h at an intensity of 30 W/m^2^—both longer and at a higher intensity compared to our treatment (30 min. at 22 W/m^2^). Yields in Micro Tom were 6.7 ± 1.73 μg/g DW in leaves, 0.38 ± 0.06 μg/g DW in green fruit and 0.27 ± 0.02 μg/g dry weight in ripe fruits (Figure 1C; Figure S2e–g). Therefore, while deletion of *7‐dr2* leads to production of vitamin D_3_, we concluded that alternative approaches to improve the levels of vitamin D_3_ in plants could be explored.

We noted that the levels of cholesterol were significantly higher in leaves of *7‐dr2* mutant lines compared to wild type, consistent with earlier reports (Figures S1b and S2c). It is likely 7‐DR1, a close homologue of 7‐DR2 that is responsible for production of brassinosteroids, may be compensating for the loss of 7‐DR2. Therefore, we hypothesized that an enzyme that could convert cholesterol back to 7‐DHC, for example a cholesterol 7‐desaturase enzyme, could mitigate this effect. A cholesterol 7‐desaturase enzyme has never been reported in any land species, but these enzymes are common in insects that convert dietary cholesterol to ecdysones (e.g. 20‐hydroxyecdysone), steroid hormones crucial for insect development. In fact, the first step in ecdysone biosynthesis is conversion of cholesterol to 7‐DHC. This irreversible step is catalysed by Neverland (NVD), an evolutionarily conserved oxygenase‐like protein (Yoshiyama *et al*., 2006, 2011). Therefore, we decided to take an advantage of this unique enzyme for vitamin D_3_ engineering by overexpressing a codon‐optimized version of NVD (synthetic gene) in the *7‐dr2* tomato mutant lines via stable genetic transformation.

We selected the silkworm *Bombyx mori* Neverland (BmNVD) enzyme that has been reported to catalyse the conversion of cholesterol to 7‐DHC (Yoshiyama *et al*., 2006, 2011). Since expression of properly folded, active insect enzymes is challenging in heterologous plant hosts, we first tested the capacity of codon‐optimized version of BmNVD enzyme in leaves of *N. benthamiana*, a substantial producer of cholesterol, by *Agrobacterium tumefaciens*‐mediated transient expression. Metabolic profiling of the leaf extracts by GC–MS showed that transient expression of BmNVD clearly led to the accumulation of 7‐DHC (Figure 1d; Figure S3). Moreover, treatment of transiently infiltrated leaves (after 3 days) with UV‐B light indeed resulted in the production of vitamin D_3_ (Figure 1d). Confocal microcopy analysis of the BmNVD:RFP infiltrated *N. benthamiana* disks showed that *BmNVD* is localized to the endoplasmic reticulum (ER) (Figure 1e). In plants, cholesterol is synthesized in the ER; therefore, co‐localization of BmNVD enzyme in the same compartment likely allows the direct access to the cholesterol substrate, facilitating its conversion to 7‐DHC. Altogether, these results demonstrate that the codon optimized BmNVD is active, and able to function normally in heterologous plant host system.

Inspired by these results, we next overexpressed *BmNVD* (codon optimized) in the *7‐dr2ko* mutant tomato (*cv*. Money Maker) plants (Figure S4a,b). The commercial variety Money Maker was selected for the appealing flavour of the fruit, which is in contrast to the Micro Tom, whose fruits are more bitter and therefore less suitable for consumption. Homozygous *7‐dr2ko* mutant lines overexpressing *BmNVD* (*BmNVDOx + 7‐dr2ko*) were further analysed for altered steroidal metabolite profiles (Figure 1F; Figure S4c,d,f,g). Though high levels of 7‐DHC were observed in leaves of *BmNVDOx + 7‐dr2ko* transgenic lines compared to WT, we still noticed the significant accumulation of cholesterol in these lines (Figure S4c,f), suggesting compensatory activity from 7‐DR1, involved in brassinosteroid biosynthesis. Subsequent UV‐B treatment of leaves and green fruits of *BmNVDOx + 7‐dr2ko* genotype resulted in ~3 and ~ 5‐fold increase in vitamin D_3_ levels, respectively as compared to the ones produced by *7‐dr2ko* mutant alone (18 ± 2.1 *Vs* 5.1 ± 1.2 μg/g DW in leaves and 0.76 ± 0.14 *Vs* 0.15 ± 0.01 μg/g DW in green fruit) (Figure 1C; Figure S4e,h). As observed earlier with the *7‐dr2ko* mutant lines, treatment of red fruits from *BmNVDOx + 7‐dr2ko* plants did not yield any quantifiable amount of vitamin D_3_. The levels of vitamin D_3_ produced in tomato waste material, for example the leaves, offers a promising source for plant‐derived vitamin D_3_ supplements and can easily cover the recommended daily intake of vitamin D_3_, which ranges between 10 and 20 μg depending mainly on age. In summary, our findings contribute to the exploration of innovative methods for vitamin D_3_ biofortification, addressing global deficiencies and improving accessibility via a sustainable, plant‐based and cost‐effective platform.

## Conflict of interest

The authors declare no conflict of interest.

## Author contributions

M.B. designed, performed the research and wrote the manuscript. M.B. and V.G. carried out confocal imaging analysis. R. K., M. H. and M. K. assisted in steroidal metabolite profiling. K.P. performed tomato transformation and regeneration. P.D.S. and S.E.O'C. conceived and designed the research project and wrote the manuscript, with contributions from all authors.

## Supporting information

Supplementary Methods.
**Figure S1–S4** Supplementary Figures.
**Table S1** List of oligonucleotides used in this study.

## Data Availability

Data supporting the findings of this work are available within the paper and its Supplementary information file.
