# Supplementary material for: Metabolic engineering of vitamin D3 in Solanaceae plants
Source: Plant Biotechnol J. 2024 Sep 16;22(12):3389–91. doi: 10.1111/pbi.14459 (PMC11606407; doi:10.1111/pbi.14459)
Supplement: Supplementary file 1 — Supplementary Methods. Figure S1–S4 Supplementary Figures. Table S1 List of oligonucleotides used in this study. [file PBI-22-3389-s001.docx]

**Supplementary Materials**

**Metabolic engineering of vitamin D3 in *Solanaceae* plants**

Marianna Boccia1, Kerstin Ploβ1, Maritta Kunert1, Radhika Keshan1, Mustafa Hatam1, Veit Grabe2, Sarah E. O’Connor1* and Prashant D. Sonawane1*

1Department of Natural Product Biosynthesis, Max Planck Institute for Chemical Ecology, D-

07745 Jena, Germany

2Microscopy Imaging service, Max Planck Institute for Chemical Ecology, D-07745 Jena,

Germany

*Corresponding author: [psonawane@ice.mpg.de](mailto:psonawane@ice.mpg.de)

[oconnor@ice.mpg.de](mailto:oconnor@ice.mpg.de)

**Supplementary Methods**

**Plant sources and growth conditions**

*S. lycopersicum* (*cv.* Money Maker and Micro Tom) were used as genetic background to generate different genotypes (e.g., *7-dr2ko*, *BmNVD+7-dr2ko*) via stable transformation experiments. Wild type (WT, non-transformed), *7-dr2* knock-out mutants and *BmNVD+7-dr2ko* genotype plants were grown in a climate controlled glasshouse at 24°C during the day and 18°C during night, with natural light at the Max Planck Institute for Chemical Ecology (MPI-CE), Jena, Germany. *Nicotiana benthamiana* seeds were germinated directly in 9x9 square containers (Goettinger) and grown in the greenhouse at 23-26°C during the day and 16-22°C during the night, with a 16/8h photoperiod for four to six weeks. For transient expression experiments, plants were transferred to walk-in growth chamber (York) with a 16/8 day/night cycle and 21°C all time at 55% humidity.

**Chemicals and solvents**

MS grade solvents for the UHPLC-MS analysis were purchased from Fisher Scientific. Methanol, ethanol, n-hexane, pentane and ethyl-acetate used for the extraction of steroidal metabolites from *N. benthamiana* and *S. lycopersicum* tissues were HPLC grade and purchased from Fisher Scientific. Analytical standards such as vitamin D3*,* cholesterol, and 7-dehydrocholesterol were purchased from Sigma-Aldrich. Kanamycin sulfate, gentamicin sulfate, rifampicin, spectinomycin were purchased from Sigma-Andrich.

**Molecular biology and cloning**

All the genes reported in this study were amplified by PCR using Phusion High-Fidelity DNA Polymerase (New England Biolabs) according to the manufacturer’s instructions. Restriction enzymes and T4 ligase (New England Biolabs) were used to clone the corresponding PCR amplicons into in-house generated plant binary vectors (3Ω1-for overexpression) (Cárdenas *et al.*, 2019), and 3Ω1:Cas9 for CRISPR knockout). Primers (listed in Table S1) were purchased from Sigma-Aldrich. *E.coli* TOP10 cells (Invitrogen) and *Agrobacterium tumefaciens* (strain GV 3101) were used for plasmid isolation and for transient expression/stable transformation in *N. benthamiana/ S. lycopersicum* plants, respectively. For plasmid isolation, Wizard Plus sv-miniprep DNA purification system (Promega) was used, while for DNA recovery from PCR gels, Zymoclean gel DNA recovery kit (ZymoResearch) was used.

**Generation of plasmids for *7-dr2* knockout (*7-dr2ko*) by CRISPR/Cas9 and overexpression of *BmNVD* by GoldenBraid cloning**

*7-dr2* knockout mutants and *BmNVD* overexpression constructs were generated using GoldenBraid cloning. Two specific sgRNA guides targeting the second and third exon of *7-dr2* were designed using CRISPR-P v2.0 (http://crispr.hzau.edu.cn/CRISPR2/) and CRISPR RGEN Tools [(http://www.rgenome.net/)](http://www.rgenome.net/). The sgRNAs (sgRNA1: CGATATCTCCTACTGGACAC and sgRNA2: TTACCTATCTCAGCCTTTGG) into an in-house developed 3Ω1 plasmid, containing the Cas9 and kanamycin-resistance-expression (nptII) cassettes byGoldenbraid cloning (Ma *et al.*, 2015; Ma and Liu, 2016; Sarrion-Perdigones *et al.*, 2013). The sgRNAs were inserted in the 3Ω1:Cas9 plasmid under the control of the *Arabidopsis thaliana* U6-26 promoter. Codon optimized *BmNVD* was synthesized from Twist Bioscience [(https://www.twistbioscience.com)](https://www.twistbioscience.com/) and further cloned into 3Ω1 plant expression vector, harboring a *SlUbi10* promoter, *SlUbi10* terminator and the kanamycin resistance (nptII) expression cassette for plant selection, using the primers listed in Table S1.

**Transient expression of *BmNVD* in *Nicotiana benthamiana* leaves**

The 3Ω1:BmNVD and 3Ω1:empty vector (EV) plasmids were transformed into *Agrobacterium*

*tumefaciens* (strain GV 3101) by electroporation*.* One-single colony of each construct was used to inoculate 10 mL LB media culture supplemented with 200 μg mL-1 spectinomycin, 100 μg mL-1 rifampicin and 50 μg mL-1 gentamycin. The bacterial cultures were incubated overnight at 28°C with shaking (200 rpm). The cultures were centrifuged at 2,000 × g for 20 min and cell pellets were washed once with 5 ml of infiltration buffer [50 mM MES buffer (pH 5.6), 10 mM MgCl2, 150 μM acetosyringone]. Finally, each pellet was resuspended in 10 mL of infiltration buffer and incubated at room temperature for 1-2 h. Optical density (OD600) was set at 0.4. *Agrobacterium* suspensions were infiltrated into 4–6-week-old *N. benthamiana* leaves. Leaves were harvested 3 day-post infiltration and underwent either UV-B irradiation for vitamin D3 production, or sterols extraction for measuring 7-DHC accumulation.

**Generation of transgenic tomato plants**

3Ω1:Cas9:*7dr2* and 3Ω1:*BmNVD* constructs were transformed separately or in combination in

tomato (*cv.* Money Maker and Micro Tom) using *Agrobacterium tumefaciens* (strain GV3101)-

mediated transformation. Cotyledons were excised from the 7 to 10-day-old tomato seedlings grown in vitro, and placed onto plates containing appropriate co-cultivation media. Explants were preincubated for 24 h at room temperature (RT) under dark conditions. Co-cultivation of excised explants with *Agrobacterium* (OD600 = 0.3) was conducted for 48 h under dark conditions. For *BmNVDOx+7-dr2ko* genotype the final OD of the mixed agrobacteria was adjusted to 0.4. After the co-cultivation period, the explants were transferred to shoot induction medium consisting of zeatin riboside (2 µg ml−1), 1-napthaleneacetic acid (NAA; 0.2 µg ml−1), Kanamycin (50 µg ml−1), and Ticarcillin (250 µg ml−1) for 3 to 8 weeks. Media was changed every 2 weeks. Subsequently, well-developed shoots wereexcised and transferred to rootingmedium containingindole-3-butyric acid (IBA; 1mg ml−1), Kanamycin (50 µg ml−1), and Ticarcillin (100 µg ml−1). After 3–4 weeks, plantlets with roots were transferred to greenhouse for further analysis. Two independent *7dr2-ko* lines (*#1, #2* for Micro Tom and *#11, #12* for Money Maker), and two independent *BmNVDOx+7-dr2ko* (*#21, #22*) lines were selected in T1 generation based on the mutations confirmed by Sanger sequencing of clones amplified using *7-DR2* target site-specific oligonucleotide pairs (F: AAATGACAGGAAAACGGAGAG; R: TAGACCAGCTTGTGTAAGTGCA)and *BmNVD* site-specific oligonucleotide pairs (F: TTCCTACTCCTTCAGCGTAACA; R: TGCAGAAACCTCAAATTCATTCCT). The genomic DNA was obtained usingthe Qiagen plant genomic DNA kit by following the manufacturer’s instructions. Mutations in the transformants were identified by analyzing the sequencing data using Geneious software. Three biological replicates (n=3) from each genotype were used for various analysis. For example, #1 and #2 are two independent 7*-dr2ko* mutant line and each mutant line represents three biological samples collected from three different plants.

**Quantitative real-time PCR analysis**

*BmNVD* gene expression analysis was performed with three biological replicates (n ≥ 3). RNA isolation was performed using the Qiagen RNasi mini plant kit following the manufacturer’s instruction. The RNA was treated with a DNase I (Sigma-Aldrich) and reverse transcribed using a cDNA reverse transcription kit (SuperScript IV Reverse Transcriptase – ThermoFischer). Gene-specific oligonucleotides weredesigned with the Primer Express 2 software (Applied Biosystems). *EF1*(*Elongation Factor 1*) genewasused as endogenous control *N. benthamiana*samples (primers are listed in Table S1) (Expósito-Rodríguez *et al.*, 2008; Zhang *et al*., 2023).

**LC-MS-based sterols extraction and analysis**

Vitamin D3, cholesterol and 7-DHC profiling was performed as described earlier by Barnkob *et al.* 2019. Briefly, 150 mg of frozen material was placed in a 2 mL Eppendorf tube and saponified over night at 20°C by mixing 100 uL of 60% KOH, 300 uL of 15% ascorbic acid and 500 uL of 96% EtOH and using as internal standard pregnenolone (3 µg mL-1). The unsaponifiable matter was extracted with 20% ethyl acetate in pentane, shaked for 30’ and centrifuged for 5’ at 2,000 x g. The organic layer was transferred in a new 2 mLEppendorf tube and the extraction was repeated again. The extracts were washed with 500 uL of 0.05 mol L-1 hydrochloric acid, and mixed gently for 30 times. The organic layer was transferred to a LC-MS flask and evaporated to dryness using a gentle stream of nitrogen at room temperature. The residue was dissolved in 150 µL of MeOH, filtered through PTFE (0.22 µm) filters and analyzed my UHPLC-MS/MS. Metabolites wereanalyzed byUHPLC-MS/MS usingaThermo ScientificUltiMate 3000 RSultra-high performance liquid chromatography (UHPLC) system (Thermo Scientific, Germering, Germany) coupled to a triple quadrupole mass spectrometer (EVOQ Elite™, Bruker Daltonics, Bremen, Germany). Chromatography was performed using a Kinetex 2.6 uM F5 column (150 mm x 2.1 mm) kept at 40 °C. Water containing 35% of methanol and 0.1% formic acid and MeOH containing 0.1% formic acid were used as mobile phases A and B, respectively with a flow rate of 0.4 mL min-1. The gradient went from 60% to 100% phase B over 8 min, held 100% B for 3 min, and from 100% to 60% phase B over 3 min returning to the initial conditions. The analysis was carried out in positive mode (ESI) and the samples were kept at 10 °C. The injection volume of both the standard solutions and the samples was 5 µL. Capillary voltage was 4000V; the source was kept at 400 °C; cone temperature was 350 °C; cone gas flow 20 L h-1; and nebulizer gas flow, 50 L h-1. The analysis was performed in MRM mode operating the Q1 mass analyzer under unit resolution (0.7 Da FWHM) and Q3 mass analyzer at 2.0 Da FWHM. MRM transitions were determined from the analytical standards and used to record during sample analysis: vitamin D3 (quantifier: m/z 385.3🡪259.2, qualifier 1: m/z m/z 385.3🡪367.3, qualifier 2: m/z 385.3🡪159.1) and 7-DHC (quantifier: m/z 367.3🡪159.1, qualifier 1: m/z 367.3🡪145.1, qualifier 2: m/z 367.3🡪105.2). The EVOQ chromatograms were analysed using Data Review version 8.2.1 of the MS workstation software (Bruker Daltonics, Bremen, Germany).

**Sterols extraction for GC-MS analysis**

Leaf of tomato and *N. benthamiana* and green unripe tomato frozen tissues (100 mg) were powdered and saponified at 65 °C for 2 h in 600 µL of 20% KOH (w/v) in 50% ethanol in a thermoshaker (400 rpm, Eppendorf ThermoMixer). Samples were extracted three times with 500 µLhexane, and the combined phases were evaporated to dryness using a gentle stream of nitrogen. The samples were resuspended in 50 µl of N-methyl-N-(trimethylsilyl) trifluoroacetamide (MSTFA), and incubated for 10 min at room temperature and then for 10 min at 65 °C. Samples were transferred to a glass inserts and or not, depending on the run conditions and analyte concentration if necessary, a sample dependent dilution was performed to be within the linear range of the detector. 1µL of final volume for each sample was injected onto GC-MS system comprised a GC PAL auto sampler (CTC Analytics), a trace 1310 GC ultra-gas chromatograph equipped with a split–splitless injector and ISQ LT quadrupole mass spectrometer (Thermo Scientific). GC was performed on a 30 m × 0.25 mm × 0.25 μm Zebron ZB-5 column with 10 m guard column (Phenomenex). Samples were analyzed in the split mode (split 1:20) and the inlet temperature was set at 250 °C. Separation of analytes was done by using the following chromatographic conditions: helium was used as carrier gas at a flow rate of 1.1 ml min-1. The thermal gradient used started at 160 °C(hold 1.5 min), ramped up to 270 °Cat 30 °C min-1 ,ramped up to 290 °C at 1 °C min-1 (hold 10 min) and then finally up to 300 °C at 30 °C min-1 (hold 2.5 min). Eluents werefragmented in the electron impact mode with an ionization voltageof 70 eV, and the mass spectrometry transfer line temperature was set at 290 °C and the ion source at 250 °C. The chromatograms and mass spectra were evaluated using Xcalibur software (v.4.2.47; Thermo Scientific). Sterol compoundswereidentified bycomparingtheir retention time and mass spectrum with those generated for authentic standards (trimethylsilylated) analyzed on the same instrument and those reported in the literature (Kamal-Eldin *et al*., 1992; Yang *et al.*, 2001). Data analysis was performed with Xcalibur software (Thermo Scientific).

**UV-B treatment**

TheUV-Btreatment was performed with a transilluminator with a wavelength of 302nm (Analytik

Jena, M-20V). Detached leaves of infiltrated *N. benthamiana* plants and detached young leaves of tomato were exposed to the UV-B light source for 30 min with an intensity at 302nm of 22 W/m2. The same parameters were used also for the treatment of green and red fruits that were previously sliced.

**Sub-cellular localization studies**

For the subcellular localization of BmNVD, the corresponding gene sequence was fused in frame

to an RFP (red fluorescent protein) marker, both under the control of SlUbi10 promoter and SlUbi10 terminator in 3Ω1-overexpression vector as described previously. An ER targeted GFP marker, were used for localization studies. Constructs were introduced in *A. tumefaciens* GV 3101 by electroporation and infiltrated in *N. benthamiana* leaves as described previously. Two days post-infiltration, micrographs were acquired from freshly punched and water embedded 5 mm leaf discs on a cLSM 880 (Zeiss, Oberkochen, Germany) with a C-Apochromat 40x/1.20 W or Plan-Apochromat 63x/1.4 Oil objective. Excitation wavelengths were generated with an Argon 488 and Helium-Neon 543 laser for GFP (8-10 % transmission, 525 PMT gain) and RFP (40-100% transmission, 750 PMT gain) respectively. The spectral detector range was set to 490-550 nm for GFP and 550-650 nm for RFP, both combined with a MBS 488/543. Pinhole was set to 1 Airy Unit and the pixel dwell time was adjusted to 1µs with an 8-fold unidirectional line averaging, controlled with ZEN black (Zeiss, Oberkochen, Germany). Pixel dimensions were optimized depending on the used objective, excitation wavelength and zoom factor. Transmitted light signal was acquired with a T-PMT in the RFP-track (T-PMT gain 250). Channels were scanned sequential to reduce crosstalk. Contrast and brightness improvement, cropping and scale bar insertion was done in Image J (Schindelin *et al*., 2012) and Photoshop.

**Supplementary references:**

Barnkob, L.L., Petersen, P.M., Nielsen, J.P., Jakobsen, J., 2019. Vitamin D enhanced pork from

pigs exposed to artificial UVB light in indoor facilities. *Eur. Food Res. Technol.* 245, 411– 418.

Cárdenas, P.D., Sonawane, P.D., Heinig, U., Jozwiak, A., Panda, S., Abebie, B., Kazachkova, Y., Pliner, M., Unger, T., Wolf, D., Ofner, I., Vilaprinyo, E., Meir, S., Davydov, O., Gal-on, A., Burdman, S., Giri, A., Zamir, D., Scherf, T., Szymanski, J., Rogachev, I., Aharoni, A., 2019. Pathways to defense metabolites and evading fruit bitterness in genus Solanum evolved through 2-oxoglutarate-dependent dioxygenases. *Nat. Commun.* 10, 5169.

Expósito-Rodríguez, M., Borges, A.A., Borges-Pérez, A., Pérez, J.A., 2008. Selection of internal control genes for quantitative real-time RT-PCR studies during tomato development process. *BMC Plant Biology* 8, 131.

Kamal-Eldin, A., Appelqvist, L.Å., Yousif, G., Iskander, G.M., 1992. Seed lipids of Sesamum indicum and related wild species in Sudan. The sterols. *Journal of the Science of Food and Agriculture* 59, 327–334.

Ma, X., Liu, Y.-G., 2016. CRISPR/Cas9-Based Multiplex Genome Editing in Monocot and Dicot Plants. *Curr. Protoc. Mol. Biol.* 115, 31.6.1-31.6.21.

Ma, X., Zhang, Q., Zhu, Q., Liu, W., Chen, Yan, Qiu, R., Wang, B., Yang, Z., Li, H., Lin, Y., Xie, Y., Shen, R., Chen, S., Wang, Z., Chen, Yuanling, Guo, J., Chen, L., Zhao, X., Dong, Z., Liu, Y.-G., 2015. A Robust CRISPR/Cas9 System for Convenient, High-Efficiency Multiplex Genome Editing in Monocot and Dicot Plants. *Mol. Plant.* 8, 1274–1284.

Sarrion-Perdigones, A., Vazquez-Vilar, M., Palací, J., Castelijns, B., Forment, J., Ziarsolo, P., Blanca, J., Granell, A., Orzaez, D., 2013. GoldenBraid 2.0: A Comprehensive DNA Assembly Framework for Plant Synthetic Biology. *Plant Physiology* 162, 1618–1631.

Schindelin, J., Arganda-Carreras, I., Frise, E., Kaynig, V., Longair, M., Pietzsch, T., Preibisch, S., Rueden, C., Saalfeld, S., Schmid, B., Tinevez, J.-Y., White, D.J., Hartenstein, V., Eliceiri, K., Tomancak, P., Cardona, A., 2012. Fiji: an open-source platform for biological-image analysis. *Nat. Methods* 9, 676–682.

Yang, B., Karlsson, R.M., Oksman, P.H., Kallio, H.P., 2001. Phytosterols in sea buckthorn (Hippophaë rhamnoides L.) berries: identification and effects of different origins and harvesting times. *J. Agric. Food Chem.* 49, 5620–5629.

Zhang, G., Zhang, Z., Wan, Q., Zhou, H., Jiao, M., Zheng, H., Lu, Y., Rao, S., Wu, G., Chen, J., Yan, F., Peng, J., Wu, J., 2023. Selection and Validation of Reference Genes for RT-qPCR Analysis of Gene Expression in Nicotiana benthamiana upon Single Infections by 11 Positive-Sense Single-Stranded RNA Viruses from Four Genera. *Plants (Basel)* 12, 857.


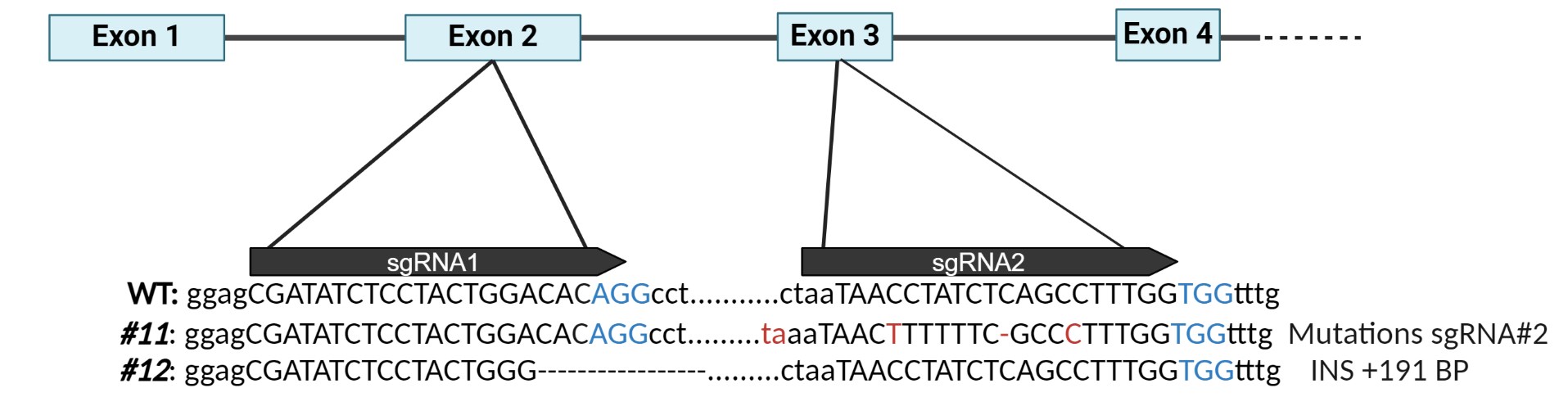
**a**

**#11**

**#12**

**WT**

**#11**

**#12**

**WT**

**Vitamin D3**

**(μg/gdry weight)**


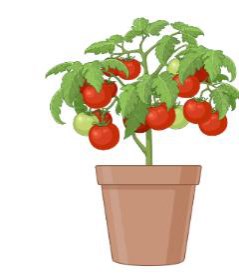
**Cholesterol** **7-DR2** **7-DHC** **UV-B** **Vitamin D3**

**b** **c**

**8×107** ** **8×108**


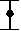


* * *

**cholesterol levels**

**(peak area)**

**6×107**


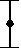


**7-DHC levels**

**(peak area)**


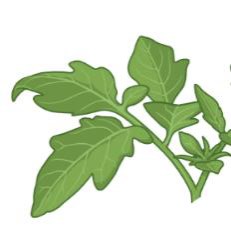
**4×108**

**4×107**


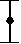


**d**

**10**


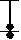

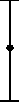


**8**

**Vitamin D3**

**(μg/gdry weight)**

**6**

**4**

**2×107** **1×105** **2**

**0** **0** **0** nd

**#11**

**#12**

**WT**

**#11**

**#12**

**WT**

**#11**

**#12**

**WT**

**e** **f** **g**

**2.5×104**

**2×104**

**cholesterol levels**

**(peak area)**


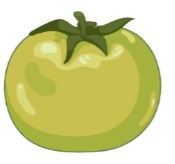
**1.5×104**

**1×104**

**5×103**

**0**

**1.5×105**

**1×105**

**7-DHC levels**

**(peak area)**

**5×104**

**0**

**0.20**

**0.15**


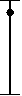

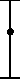


**0.10**

**0.05**

nd **0.00**

**#11**

**#12**

**WT**

**Figure S1: Knockout of *7-dr2* in tomato (*cv.* Money Maker) plants leads to the**

**accumulation of 7-DHC and vitamin D3 (upon UV-B treatment). (a)** Schematic representation of *7-DR2* genomic sequence with location of guide RNAs and *7-dr2ko*

mutant sequences. Two independent *7-dr2ko* mutant lines were generated *(#11, #1*2).

Protospacer adjacent motifs (PAMs) and inserted nucleotides are marked in blue and red

respectively, while deleted nucleotides are replaced by dashes. **(b-g)** Levels of cholesterol

(b, e), 7-DHC (c, f) and vitamin D3 (d, g) in leaves (b-d) and green fruits (e-g) of wild-type (WT) and *7-dr2ko* mutant lines as determined by GC-MS or LC-MS. The values

indicate means of biological replicates ± standard error (n=3). Nd: not detected. Statistical

significance of cholesterol levels in mutant lines compared to WT was assessed using two-

tailed t-tests (**P*≤ 0.05, ***P*≤ 0.005).

**a** **b**


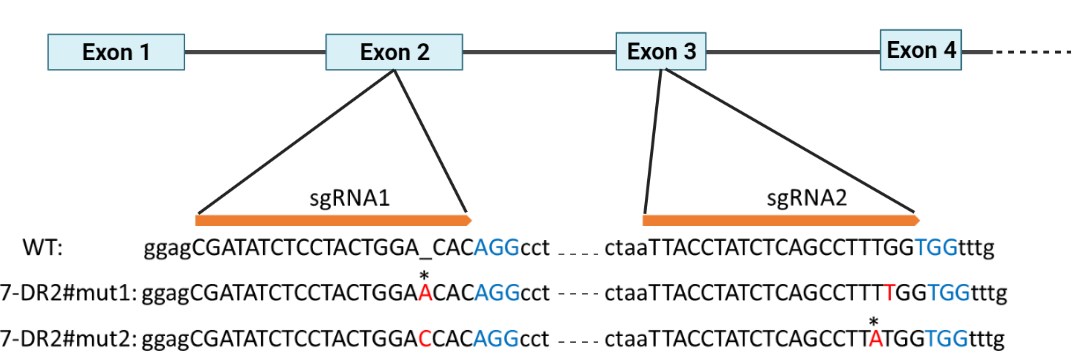


**#1**

**#2**

**WT**

**#1**

**#2**

**WT: *#1*:**


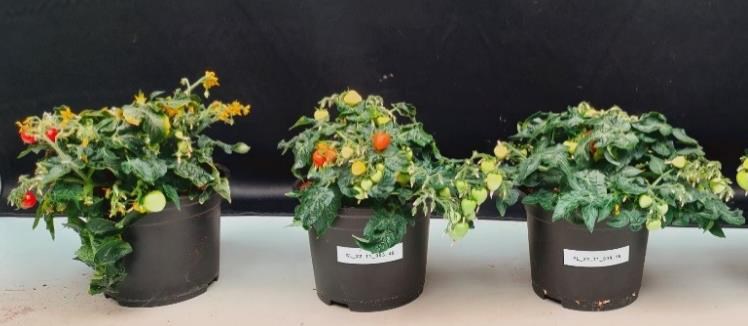


***#2*:** MicroTom WT 7dr2ko#1 7dr2ko#2


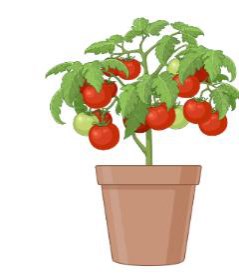
**Cholesterol** **7-DR2** **7-DHC** **UV-B** **Vitamin D3**

**c**

**2×107**


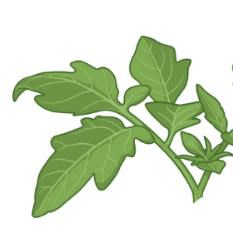
**1.5×107**

**cholesterol levels**

**(peak area)**

**1×107**

**5×106**

**0**

**d** **e 5×107 15**

**3×107** **10**

**7-DHC levels**

**(peak area)**

**Vitamin D3**

**(μg/gdry weight)**

**1×107**

**100000** **5**

**50000**

**0** **0**

**#1**

**#2**

**WT**

nd


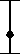

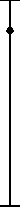


**WT**


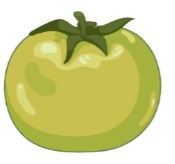

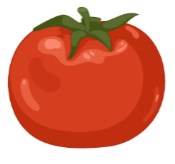
**f** **g**

**0.8** **0.4**

**0.6** **0.3**

**Vitamin D3**

**(μg/gdry weight)**

**Vitamin D3**

**(μg/gdry weight)**

**0.4** **0.2**

**0.2** **0.1**

**0.0** nd **0.0** nd

**#1**

**#2**

**WT**

**#1**

**#2**

**WT**

**Figure S2: Knockout of *7-dr2* in *S. lycopersicum cv*. Micro Tom plants. (a)** Schematic

representation of *7-DR2* genomic sequence with location of guide RNAs and *7-dr2ko*

mutant sequences. Two independent *7-dr2ko* mutant lines *(#1, #2)* were generated.

Protospacer adjacent motifs (PAMs) and inserted nucleotides are marked in blue and red

respectively, while deleted nucleotides are replaced by dashes. **(b)** Comparison of WT and

*7-dr2ko* mutant tomato plants. *7-dr2* knockout did not effect the normal growth phenotype

of the plants. **(c-e)** Levels of cholesterol **(c)**, 7-DHC **(d)** and vitamin D3 **(e)** in leaves of WT and *7-dr2ko* mutant lines as determined by GC-MS or LC-MS. **(f, g)** Levels of vitamin

D3 in green **(f)** and red **(g)** fruit of *7-dr2ko* mutant lines. The values indicate means of biological replicates ± standard error (n=3). Nd: not detected.


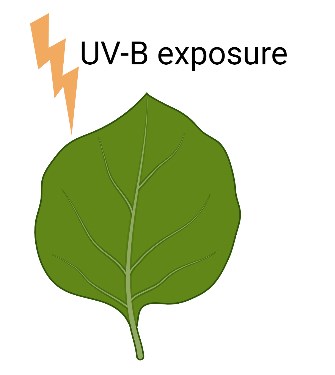
**1.5×106**


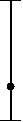


***BmNVD* relative transcript**

**1×106**

*N. benthamiana* **5×105** overexpressing *BmNVD*

**0**

**BmNVD**

**EV**

**WT**

**Figure S3: Expression of *Bombyx mori Neverland* (*BmNVD*) in transiently infiltrated**

***N. benthamiana* leaves as determined by qRT-PCR.** As EV and WT samples did not

show any detectable *NVD* expression levels, these samples were considered with a

expression value of 0.001 for relative quantification of *NVD* in *BmNVD* samples.


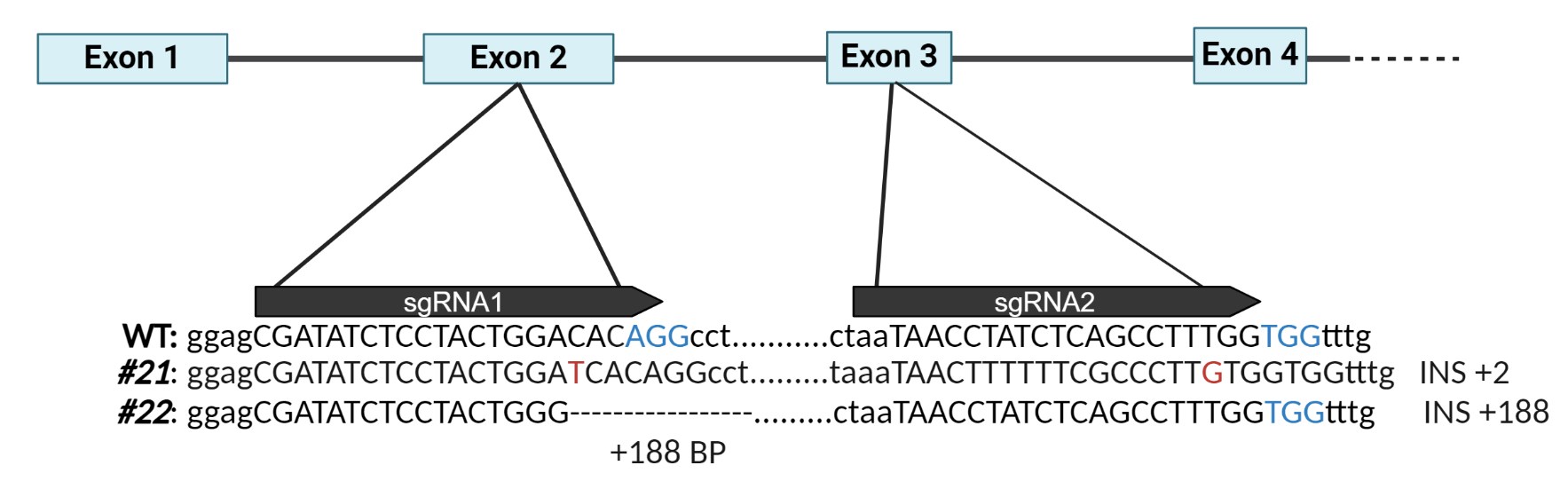

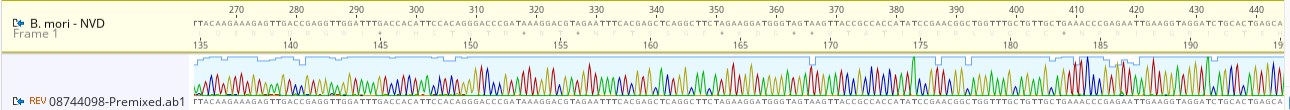
**a**

**b**

***#21***: Genotyping of *BmNVD*


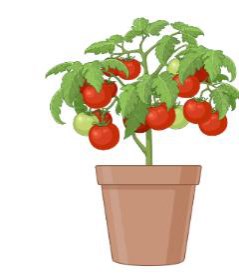

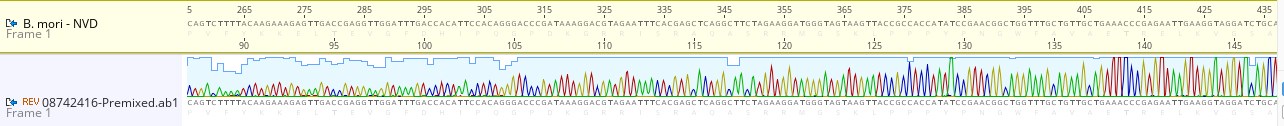
***#22***: Genotyping of *BmNVD*

**7-DR2 Cholesterol** **NVD** **7-DHC**

**UV-B** **Vitamin D3**

**c** **d** **e**

**1.2×108** * * **1×109 30 25**


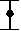

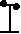

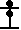

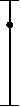

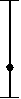

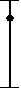


**cholesterol levels**

**(peak area)**

**7-DHC levels**

**(peak area)**

**Vitamin D3**

**(μg/gdry weight)**


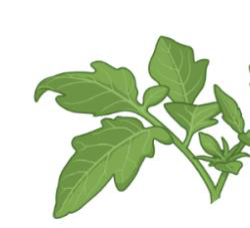
**8×107** **5×108** **20**


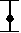

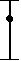


**15 4×107** **10**

**1×107** **1×105** **5**

**0** **0** **0** nd

**#21**

**#22**

**WT**

**#21**

**#22**

**WT**

**#21**

**#22**

**WT**

**f**

**2.5×104**

**g** **h**

**8×105** **1.5**


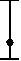

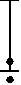


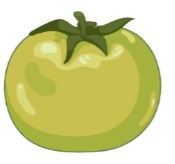
**2×104** **6×105**

**cholesterol levels**

**(peak area)**

**7-DHC levels**

**(peak area)**

**Vitamin D3**

**(μg/gdry weight)**

**1.5×104** **1.0**

**4×105**

**1×104** **0.5 5×103 2×105**

**0** **0** **0.0** nd

**#21**

**#22**

**WT**

**#21**

**#22**

**WT**

**#21**

**#22**

**WT**

**Figure S4: Engineering high vitamin D3 levels in tomato (*cv.* Money Maker) by combinatorial strategy involving knockout of *7-dr2* and overexpression of *BmNVD*.**

**(a)** Schematic representation of *7-DR2* genomic sequence with location of guide RNAs

and *7-dr2ko* mutant sequences. Two independent *7-dr2ko+BmNVD* mutant lines *(#21,*

*#22)* were generated. Protospacer adjacent motifs (PAMs) and inserted nucleotides are

marked in blue and red respectively, while deleted nucleotides are replaced by dashes. **(b)**

Representative *BmNVD* genotyping in double mutant lines**. (c-h)** Levels of cholesterol (c,

f), 7-DHC (d, g) and vitamin D3 (e, h) in leaves (c-e) and green fruits (f-h) of WT and

*7-dr2ko+BmNVD* genotype lines. The values indicate means of biological replicates ± standard error (n=3). Nd: not detected. Statistical significance was calculated using two-tailed t-tests (**P*≤ 0.05, ***P*≤ 0.005). Refer Figure 1C for summary of average vitamin D3 levels generated in different genotypes.

**Table S1.** List of oligonucleotides used in this study

Gene Oligo Sequence Explanation

F

TTCAGAGGTCTCTCTCGATGGCTGACAGACAGCACTTC

BmNVD R

In 3Ω1 plant binary vector for transient and stable

**BmNVD**:RFP

BmNVD:**RFP**

BmNVD

BmNVD

7-DR2

7-DR2

AGCGTGGGTCTCG ACCG TTACCAATCTAACGGATTTTG F TTCAGAGGTCTCTCTCGAATGGCTGACAGACAGCACTTC R AGCGTGGGTCTCGCGTCCCAATCTAACGGATTTTGAA

F TTCAGAGGTCTCTGACGGGATGGCCTCCTCCGAGGACGTCA T

R AGCGTGGGTCTCGACCGTCAGGCGCCGGTGGAGTGGCG F

TTTGCTGTTGCTGAAACCCG R TCAACGACACACCACGTTCT

F

TTCCTACTCCTTCAGCGTAACA R

TGCAGAAACCTCAAATTCATTCCT F TGCTGATGGATCTGTATTGCAA

R

ACTCCATCCCCCAATAGTAGTCA

F CGATATCTCCTACTGGACACGTTTTAGAGCTAGAAAT

R GTGTCCAGTAGGAGATATCGTGACCAATGTTGCTCC

F TTACCTATCTCAGCCTTTGGGTTTTAGAGCTAGAAAT

R CCAAAGGCTGAGATAGGTAATGACCAATGGTGCTTTG

transformations

In 3Ω1 plant binary vector for sub-cellular

localization

qPCR analysis

*S. lycopersicum* Genotyping of stable transgenic lines

*S. lycopersicum* Genotyping of stable transgenic lines

CRISPR/Cas9 plasmid for stable *S. lycopersicum* stable transformation

F

7-DR2 R

TGTGCTATGGTATGGACTATGGA

GTTTACCCGCCAATATATCCTGTC

Sequencing CRISPR/Cas9 plant binary vector
